# Draft Genome Sequence and Annotation of the Entomopathogenic Bacterium *Xenorhabdus nematophila* Strain F1

**DOI:** 10.1128/genomeA.00342-13

**Published:** 2013-06-20

**Authors:** Anne Lanois, Jean-Claude Ogier, Jérome Gouzy, Christine Laroui, Zoé Rouy, Alain Givaudan, Sophie Gaudriault

**Affiliations:** Diversité, Génomique et Interactions Microorganismes-Insectes (DGIMI) Institut National de la Recherche Agronomique, Montpellier, Francea; Diversité, Génomique et Interactions Microorganismes-Insectes (DGIMI), Université Montpellier 2, Montpellier, Franceb; Laboratoire des Interactions Plantes Microorganismes (LIPM)–UMR441/2954 Institut National de la Recherche Agronomique, Centre National de la Recherche Scientifique, Castanet Tolosan, Francec; CEA, Genoscope, CNRS-UMR 8030, Laboratoire d’Analyse Bioinformatique en Génomique et Métabolisme, Evry, Franced

## Abstract

We report the 4.3-Mb genome sequence of *Xenorhabdus nematophila* strain F1, a Gram-negative bacterium that is a symbiont of the entomopathogenic nematode *Steinernema carpocapsae* and pathogenic by direct injection for a wide variety of insects.

## GENOME ANNOUNCEMENT

The *Xenorhabdus* genus belongs to the *Enterobacteriaceae* family (1). It contains species that are symbionts of nematodes of the family *Steinernematidae*, pathogenic for a wide variety of insects. The entomopathogenic nematodes are used as effective biological control agents for soil-inhabiting insects and they have been commercially available since the 1970s (2). Bacteria alone may also be pathogenic for insects. The entomopathogenic bacteria are good models for deciphering both pathogenic and mutualistic interaction with invertebrates. The studies of the molecular mechanisms governing host-bacterium interactions are mainly achieved in the *Xenorhabdus nematophila* species (1, 3). The genome of the type strain *X. nematophila* ATCC 19061, isolated from an American (United States) nematode *Steinernema carpocapsae*, has been recently sequenced and analyzed (4–6). The *X. nematophila* strain F1, isolated from the nematode *Steinernema carpocapsae* “Plougastel” from Brittany, France, was also highly genetically documented (for example, see references 7–17), but no genomic data were available. Therefore, we sequenced the genome of this highly studied strain.

The genomic DNA of *X. nematophila* strain F1 was purified from our laboratory stock according to the method of Brenner et al. (18). Sequencing with the Illumina HiSeq 2000 sequencer resulted in 24,259,054 single-end reads with a length of 36 nucleotides (performed by Montpellier MGX genomix, Montpellier, France) and in 60,622,006 mate-pair reads of a 3-kb insert size library with a length of 50 nucleotides (performed by GATC Biotech AG, Konstanz, Germany). Single-end reads were contiged using SOAPdenovo 1.05 (19), and then contigs were scaffolded with LYNX (J. Gouzy, unpublished data) by utilizing mate-pair information. Final assembly consisted of 61 scaffolds with 351 contigs comprising a total length of 4.3 Mb (4.2 Mb without undetermined bases). The assembly has an N_50_ scaffold size of 401,015 nucleotides and a GC content of 43.64%. Functional annotation was carried out using tools of the MicroScope platform (20), and the annotated genome was implemented in the public XenorhabduScope database (https://www.genoscope.cns.fr/agc/microscope/home/index.php). The assembly contains 4,325 genomic objects, among which are 4,245 coding sequences, 3 rRNA genes, 47 tRNA genes, and 30 noncoding RNAs.

3,716 coding sequences (CDS) (88% of the whole CDS) of the *X. nematophila* F1 genome are also present in *X. nematophila* ATCC 19061 and are considered the *X. nematophila* species core genome. Among the 529 CDS specific to *X. nematophila F*1, 420, 45, and 3 are annotated as proteins of unknown function, phagic proteins, and transposases, respectively. The remaining CDS are mainly annotated as involved in metabolism, transport, and plasmid mobilization. The genome information provided here will allow for genomic experiments such as RNA-Seq and ChIP-Seq analysis on the *X. nematophila* F1 strain.

### Nucleotide sequence accession numbers.

This whole-genome sequence of *Xenorhabdus nematophila* strain F1 has been deposited at the European Nucleotide Archive under the accession number CAVM000000000. The version described is accession no. CAVM000000000.1.

## References

[1] Nielsen-LeRoux C Gaudriault S Ramarao N Lereclus D Givaudan A 2012 How the insect pathogen bacteria *Bacillus thuringiensis* and *Xenorhabdus*/*Photorhabdus* occupy their hosts. Curr. Opin. Microbiol. 15:220–231 22633889 10.1016/j.mib.2012.04.006

[2] Ehlers RU 2001 Mass production of entomopathogenic nematodes for plant protection. Appl. Microbiol. Biotechnol. 56:623–633 11601608 10.1007/s002530100711

[3] Richards GR Goodrich-Blair H 2009 Masters of conquest and pillage: *Xenorhabdus nematophila* global regulators control transitions from virulence to nutrient acquisition. Cell. Microbiol. 11:1025–1033 19374654 10.1111/j.1462-5822.2009.01322.x PMC2811582

[4] Latreille P Norton S Goldman BS Henkhaus J Miller N Barbazuk B Bode HB Darby C Du Z Forst S Gaudriault S Goodner B Goodrich-Blair H Slater S 2007 Optical mapping as a routine tool for bacterial genome sequence finishing. BMC Genomics 8:321 17868451 10.1186/1471-2164-8-321 PMC2045679

[5] Ogier J-C Calteau A Forst S Goodrich-Blair H Roche D Rouy Z Suen G Zumbihl R Givaudan A Tailliez P Médigue C Gaudriault S 2010 Units of plasticity in bacterial genomes: new insight from the comparative genomics of two bacteria interacting with invertebrates, *Photorhabdus* and *Xenorhabdus*. BMC Genomics 11:568 20950463 10.1186/1471-2164-11-568 PMC3091717

[6] Chaston JM Suen G Tucker SL Andersen AW Bhasin A Bode E Bode HB Brachmann AO Cowles CE Cowles KN Darby C De Léon L Drace K Du Z Givaudan A Herbert Tran EE Jewell KA Knack JJ Krasomil-Osterfeld KC Kukor R Lanois A Latreille P Leimgruber NK Lipke CM Liu R Lu X Martens EC Marri PR Médigue C Menard ML Miller NM Morales-Soto N Norton S Ogier J-C Orchard SS Park D Park Y Qurollo BA Sugar DR Richards GR Rouy Z Slominski B Slominski K Snyder H Tjaden BC van der Hoeven R Welch RD Wheeler C Xiang B Barbazuk B Gaudriault S Goodner B Slater SC Forst S Goldman BS Goodrich-Blair H 2011 The entomopathogenic bacterial endosymbionts *Xenorhabdus* and *Photorhabdus*: convergent lifestyles from divergent genomes. PLoS One 6:e27909 22125637 10.1371/journal.pone.0027909 PMC3220699

[7] Givaudan A Lanois A Boemare N 1996 Cloning and nucleotide sequence of a flagellin encoding genetic locus from *Xenorhabdus nematophilus*: phase variation leads to differential transcription of two flagellar genes (fliCD). Gene 183:243–253 8996114 10.1016/s0378-1119(96)00452-0

[8] Thaler JO Duvic B Givaudan A Boemare N 1998 Isolation and entomotoxic properties of the Xenorhabdus nematophilus F1 lecithinase. Appl. Environ. Microbiol. 64:2367–2373 9647801 10.1128/aem.64.7.2367-2373.1998 PMC106397

[9] Givaudan A Lanois A 2000 flhDC, the flagellar master operon of *Xenorhabdus nematophilus*: requirement for motility, lipolysis, extracellular hemolysis, and full virulence in insects. J. Bacteriol. 182:107–115 10613869 10.1128/jb.182.1.107-115.2000 PMC94246

[10] Volgyi A Fodor A Forst S 2000 Inactivation of a novel gene produces a phenotypic variant cell and affects the symbiotic behavior of *Xenorhabdus nematophilus*. Appl. Environ. Microbiol. 66:1622–1628 10742251 10.1128/aem.66.4.1622-1628.2000 PMC92032

[11] Brillard J Ribeiro C Boemare N Brehélin M Givaudan A 2001 Two distinct hemolytic activities in *Xenorhabdus nematophila* are active against immunocompetent insect cells. Appl. Environ. Microbiol. 67:2515–2525 11375158 10.1128/AEM.67.6.2515-2525.2001 PMC92902

[12] Sicard M Brugirard-Ricaud K Pagès S Lanois A Boemare NE Brehélin M Givaudan A 2004 Stages of infection during the tripartite interaction between *Xenorhabdus nematophila*, its nematode vector, and insect hosts. Appl. Environ. Microbiol. 70:6473–6480 15528508 10.1128/AEM.70.11.6473-6480.2004 PMC525208

[13] Goetsch M Owen H Goldman B Forst S 2006 Analysis of the PixA inclusion body protein of *Xenorhabdus nematophila*. J. Bacteriol. 188:2706–2710 16547059 10.1128/JB.188.7.2706-2710.2006 PMC1428424

[14] Vigneux F Zumbihl R Jubelin G Ribeiro C Poncet J Baghdiguian S Givaudan A Brehélin M 2007 The xaxAB genes encoding a new apoptotic toxin from the insect pathogen *Xenorhabdus nematophila* are present in plant and human pathogens. J. Biol. Chem. 282:9571–9580 17229739 10.1074/jbc.M604301200

[15] Lanois A Jubelin G Givaudan A 2008 FliZ, a flagellar regulator, is at the crossroads between motility, haemolysin expression and virulence in the insect pathogenic bacterium Xenorhabdus. Mol. Microbiol. 68:516–533 18383616 10.1111/j.1365-2958.2008.06168.x

[16] Gualtieri M Aumelas A Thaler J-O 2009 Identification of a new antimicrobial lysine-rich cyclolipopeptide family from *Xenorhabdus nematophila*. J. Antibiot. 62:295–302 19373275 10.1038/ja.2009.31

[17] Jubelin G Pagès S Lanois A Boyer M-H Gaudriault S Ferdy J-B Givaudan A 2011 Studies of the dynamic expression of the Xenorhabdus FliAZ regulon reveal atypical iron-dependent regulation of the flagellin and haemolysin genes during insect infection. Environ. Microbiol. 13:1271–1284 21332625 10.1111/j.1462-2920.2011.02427.x

[18] Brenner DJ McWhorter AC Knutson JK Steigerwalt AG 1982 Escherichia vulneris: a new species of *Enterobacteriaceae* associated with human wounds. J. Clin. Microbiol. 15:1133–1140 7107843 10.1128/jcm.15.6.1133-1140.1982 PMC272265

[19] Li R Zhu H Ruan J Qian W Fang X Shi Z Li Y Li S Shan G Kristiansen K Li S Yang H Wang J Wang J 2010 De novo assembly of human genomes with massively parallel short read sequencing. Genome Res. 20:265–272 20019144 10.1101/gr.097261.109 PMC2813482

[20] Vallenet D Belda E Calteau A Cruveiller S Engelen S Lajus A Le Fèvre F Longin C Mornico D Roche D Rouy Z Salvignol G Scarpelli C Thil Smith AA Weiman M Médigue C 2013 Microscope—an integrated microbial resource for the curation and comparative analysis of genomic and metabolic data. Nucleic Acids Res. 41:D636–D647 23193269 10.1093/nar/gks1194 PMC3531135

